# Improving high throughput manufacture of laser-inscribed graphene electrodes via hierarchical clustering

**DOI:** 10.1038/s41598-024-57932-z

**Published:** 2024-04-05

**Authors:** Hanyu Qian, Geisianny Moreira, Diana Vanegas, Yifan Tang, Cicero Pola, Carmen Gomes, Eric McLamore, Nikolay Bliznyuk

**Affiliations:** 1 Department of Agricultural and Biological Engineering, University of Florida, Gainesville, FL 32611 USA; 2 Department of Agricultural Sciences, Clemson University, Clemson, SC 29634 USA; 3 Environmental Engineering and Earth Sciences Department of Engineering, Clemson University, Clemson, SC 29634 USA; 4 Department of Plant and Environmental Science, Clemson University, Clemson, SC 29634 USA; 5 Department of Mechanical Engineering, Iowa State University, Ames, IA 50011 USA; 6 Departments of Statistics, Biostatistics and Electrical and Computer Engineering, University of Florida, Gainesville, FL 32611 USA

**Keywords:** Quality control, Sensors and probes

## Abstract

Laser-inscribed graphene (LIG), initially developed for graphene supercapacitors, has found widespread use in sensor research and development, particularly as a platform for low-cost electrochemical sensing. However, batch-to-batch variation in LIG fabrication introduces uncertainty that cannot be adequately tracked during manufacturing process, limiting scalability. Therefore, there is an urgent need for robust quality control (QC) methodologies to identify and select similar and functional LIG electrodes for sensor fabrication. For the first time, we have developed a statistical workflow and an open-source hierarchical clustering tool for QC analysis in LIG electrode fabrication. The QC process was challenged with multi-operator cyclic voltammetry (CV) data for bare and metalized LIG. As a proof of concept, we employed the developed QC process for laboratory-scale manufacturing of LIG-based biosensors. The study demonstrates that our QC process can rapidly identify similar LIG electrodes from large batches (n ≥ 36) of electrodes, leading to a reduction in biosensor measurement variation by approximately 13% compared to the control group without QC. The statistical workflow and open-source code presented here provide a versatile toolkit for clustering analysis, opening a pathway toward scalable manufacturing of LIG electrodes in sensing. In addition, we establish a data repository for further study of LIG variation.

## Introduction

The unique properties of graphene and other nanocarbon allotropes facilitate diverse use in electronics, energy storage, optical systems, and sensors, among others. Defects within the unique atomic structure of graphene are known to increase the chemical reactivity, which is an important property for applications of graphene/graphitic materials^1^. In these types of carbon materials, reduction of oxide groups is often necessary for electrical applications such as chemical/biological sensors. Among the many techniques used to reduce these carbon allotropes, laser treatment is one of the most useful. Early efforts in laser fabrication of graphene devices utilized top-down approaches to modify graphene oxide powders or pastes using pulsed laser annealing^2^. These top-down fabrication techniques depend on modification of free-standing carbon films, requiring numerous manufacturing steps with various degrees of reproducibility. Fabrication of graphene electronics was significantly improved by the development of laser-inscribed graphene (LIG) by the Tour group^3^, also called laser-induced graphene.

LIG is a flexible carbon-based material that is fabricated in a one-step process. The specific laser and type of substrate significantly impact the composition of the graphitized material^4–6^. In general, the inscribed surface commonly contains porous material made up of turbostratic graphene and other carbon allotropes^4^. In addition to substrate type, laser power and mode of laser pulsing are known to greatly alter the material properties of LIG^7^. Recent efforts have focused on studying these variables as a means to control the material properties of LIG for various flexible electronic applications^8^.

LIG was originally used for development of graphene supercapacitors^9–11^. Since this original discovery, many different devices have been developed, including air filters, water treatment systems, pressure sensors, wearable electronic devices, and biosensors^8,12–15^. Among these, Muzyka and Xu^16^ conduct a quantitative and highly detailed analysis of the structure/function properties of this material. Key conclusions from this comparative study include two key results: i) sheet resistance of LIG is lower than other carbon/nanocarbon materials (reduced graphene oxide, inkjet-printed graphene, graphite oxide films), ii) laser power and contact time are critical control parameters affecting both physical properties (porosity, thickness) and electrochemical behavior (sheet resistance, electroactive surface area, capacitance, quality of sp^2^ carbon).

After the photothermal conversion of polyimide to LIG, the 3D-structured carbon material can be modified in multiple ways for applications in electroanalytical sensing. Examples of post-manufacture modification techniques for sensing applications include: (i) tuning interaction chemistry by controlling hydrophobicity^17^; (ii) metallization with gold^18^, platinum^19^, copper^20,21^, or zinc oxide^22^; (iii) modification with macrotetrolide antibiotics^23^ or synthetic ionophores^24^; or (iv) biofunctionalization with enzymes^19,25^, antibodies^26^, and aptamers^27^. Beyond sensor development, LIG has been extended for use in wearable electronics^28^, microfluidic analytical devices^29^, or cell culture assays^29^, for example. Since the onset of the pandemic, at least 12 biosensors have been developed and published using LIG as a platform^5,30–36^, which demonstrates the scalable potential of LIG in sensing^37^.

These exciting advancements using LIG as a platform for electroanalytical sensing set the stage for studies that focus on scalable manufacturing. In most cases, LIG is synthesized using polyimide films as the base material, but other substrates have been investigated^38^. Optimization of laser settings for graphitization of polyimide films has been investigated in detail for one of the most common laser instruments^7^, which led to development of best practices and protocols that aim to enhance reproducibility of LIG as a sensor platform. However, to date there is limited data on the large-scale production of LIG electrodes for analytical sensing. One key manufacturing aspect is batch-to-batch variation during carbonization of the polyimide to LIG. In our study, the batch-to-batch variation refers to not only the variability between sets of electrodes fabricated at different moments but also the potential device-to-device difference in one study.

Batch-to-batch LIG variation during the manufacturing process induces uncertainty in the electrode behavior. Unfortunately, this uncertainty is infeasible to track throughout the manufacturing process for optimizing consistency. Additionally, biosensor/chemosensor development often includes numerous post-fabrication processes that further add uncertainty. Examples of post-LIG carbonization material modification include metallization, biofunctionalization, electrolyte conditioning, or cell immobilization, among others. Taken together, it is nearly impossible to trace the source of device variability to a particular stage of the manufacturing process, implying that detailed studies of each stage are required for robust manufacturing.

Clustering analysis is a prevalent technique in quality control (QC), extensively employed for categorizing sample subtypes based on their similarities and identifying outliers^39,40^. Some widely used clustering analysis methods included k-means and hierarchical clustering, mean shift algorithm, nearest-neighbors chain algorithm, etc.^41^. The hierarchical clustering algorithm, which offers the advantage of being easy to understand and implement, does not require a pre-specified number of clusters or initial configuration assignment^42^. While these techniques (and others) are generally known in the QC literature for powdered graphene synthesis^43,44^, to our knowledge there are no studies which study QC processes in the manufacturing of LIG. Given the high throughput aspect of LIG fabrication and potential for development in any laboratory with a laser engraving instrument, it is critical to establish QC methodologies for advancing LIG manufacturing.

For the first time, we develop in this manuscript a statistical workflow and an open-source hierarchical clustering toolkit for performing QC analysis in LIG fabrication for electrochemical sensing. Cyclic voltammetry (CV) was applied to test the functionality of LIG electrodes after polyimide graphitization. An analytical tool was then developed for analysis of CV data, focusing primarily on voltammogram shapes, cathodic peak current, and charge density using ferrocyanide as a redox molecule. Together, the rapid screening via CV and automated statistical workflow represent a high throughput QC fabrication process. The aim is to select high quality LIG electrodes which have been characterized using statistical similarity and optimal functionality. In this study, manufactured electrochemical biosensors based on selected LIG demonstrate reduced sensor-to-sensor variation (high repeatability). The open-source code we developed (available in R on GitHub) has been used to analyze data collected from different operators and different labs, which is practically appealing for the purpose of automating the LIG fabrication workflow.

## Methodology

### Quality control overview

**Figure 1:**
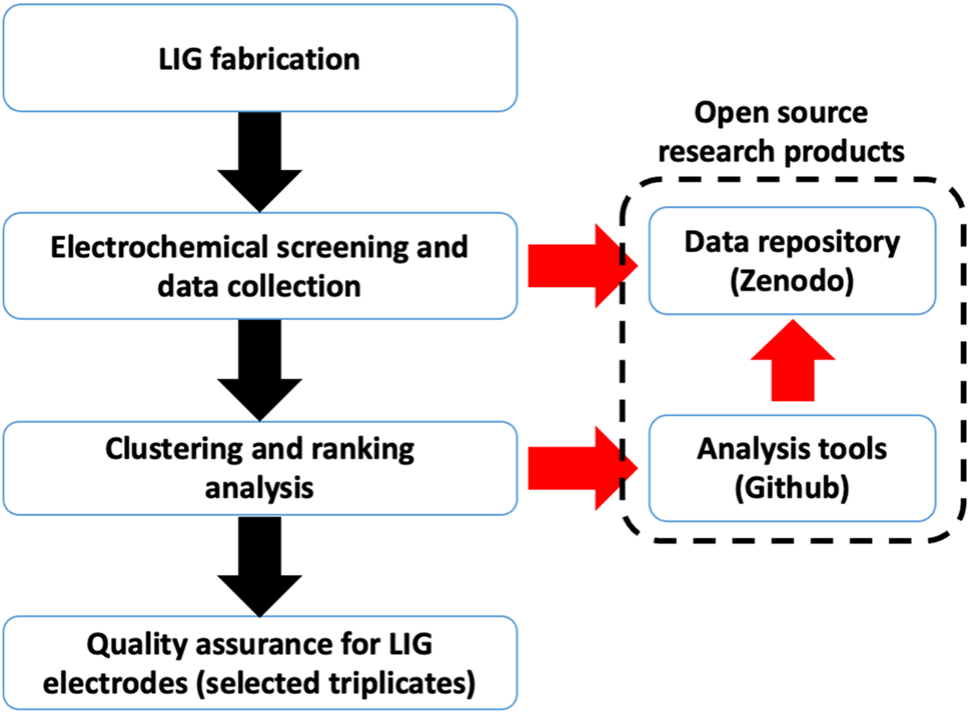
Quality control (QC) process for improving scalable manufacturing of LIG electrodes used in electrochemical sensing. An open-source data repository (Zenodo community, GitHub) includes all code, data, and additional information.

The devised QC process consists of two components: (i) electrochemical characterization of LIG electrodes, and (ii) clustering/ranking analysis (see Fig. 1). Characteristic curves obtained from CV testing were utilized to assess the performance of the electrodes. Although other QC processes in nanocarbon fabrication have used specialized techniques such as Raman spectroscopy and nanoscale metrology^43,44^, here we focus on using one of the most common techniques employed in electrochemistry. CV is an electrochemical technique which can be employed for any redox couple in solution, and a single scan be conducted in less than one minute. Although not utilized here, techniques such as fast scan CV^45^ can significantly increase the throughput. Analysis of voltammograms from CV can be simple (as is the case herein), but the data also contain a wealth of additional material related to process kinetics and reaction rate(s). For the QC process herein, we hypothesized that bare LIG electrodes exhibiting a similar shape in the CV curve demonstrated comparable electrochemical behavior when employed later in the downstream manufacturing of a biosensor, ultimately resulting in reduced device-to-device variability. Moreover, for LIG electrodes which displayed similar CV curve features (e.g., curve shape indicative of pseudo-capacitor behavior) two specific parameters were selected as response variables: i) area between the anodic and cathodic curve (representing surface charge density), and ii) peak cathodic current (representing charge transfer). It was assumed that when these two response variables were relatively high under the same test condition, improved electroanalytical signal-to-noise ratio was obtained, implying decreased random noise in the signal.

Based on the CV curves, we developed a statistical workflow to analyze the CV data which aimed to provide the information for electrode selection. The statistical workflow served as the second component of the QC process and consisted of two steps. In the initial step, the performance of each electrode was assessed individually to ascertain its proper functioning. This pretest aimed to determine whether each individual LIG working electrode met the established criteria where the electrodes were expected to achieve stable output (less than 5% deviation in voltammogram shapes) after being subjected to several successive CV sweeps. Electrodes that failed to pass this pretest were categorized as unqualified and subsequently discarded. In the subsequent step, electrodes that successfully passed the pretest underwent a selection analysis to identify electrodes that exhibited similar and good characteristics, see subsection on analysis of voltammograms for more details. The statistical workflow aims to partition the manufactured electrodes into distinct clusters based on the similarity of voltammograms, and also to identify clusters with high surface charge density and peak oxidative current. The software used in this QC process, including the ad-hoc electroanalytical software used for CV testing and biosensor signal acquisition, and statistical code for data analysis, is included in Supplementary Materials S5.

### Materials and reagents

Kapton (polyimide) film (electrical grade polyimide film, 0.005″ thick) was obtained from McMaster-Carr (Elmhurst, IL, United States). Potassium ferrocyanide [K_4_Fe(CN)_6_] and potassium ferricyanide [K_3_Fe(CN)_6_] were obtained from Thermo Fisher Scientific (Waltham, MA, United States). Potassium chloride (KCl), chloroplatinic acid solution (H_2_PtCl_6_), and lead (II) acetate trihydrate [Pb(CH_3_CO_2_)_2_·3H_2_O] were purchased from Sigma Aldrich (St. Louis, MO, United States). Ag/AgCl (3 M KCl) reference electrode (Catalog number: MF-2056) and platinum auxiliary electrode (7.5 cm, Catalog number: MW-1032) were obtained from BASi® (West Lafayette, IN, United States).

### LIG electrode fabrication

LIG working electrodes were designed in CorelDraw consisting of a circular working area (3.0 mm) connected to a stem (14.3 × 2 mm) that leads to a bonding pad area (2.9 × 2.5 mm) based on previous work^19,46^. In summary, LIG electrodes were engraved on Kapton film using a CO_2_ laser (VLS2.30DT, Universal Laser Systems, Inc., Scottsdale, AZ, US) at 75% speed, 40% power, and 1000 PPI. A passivation layer of nitrocellulose lacquer was applied to the stem area, and a metallic tape was incorporated into the bonding pad area of the working electrode. Platinum nanoparticles (nPt) were coated on the working electrode via electrodeposition by immersing the working electrode in a solution of 1.44% (v/v) chloroplatinic acid and 0.002% (v/v) lead acetate and applying a constant potential of 10 V for 90 s, as described by Moreira et al. (2022).

### Cyclic voltammetry

Electrochemical analysis of LIG electrodes was performed using CV as described by^47^. CV testing was carried out in a solution of 100 mM KCl, 2.5 mM K_3_[Fe(CN)_6_], and 2.5 mM K_4_[Fe(CN)_6_] with a potential range from − 0.8 to 0.8 V and a scan rate of 200 mV/s for ten repetitive sweeps. A benchtop MultiPalmSens4 potentiostat (PalmSens, Houten, Netherlands) connected to a 3-electrode cell stand (BASi®, West Lafayette, IN, United States) was used for all testing. The three-electrode system consisted of an Ag/AgCl (3 M KCl) as a reference electrode, platinum wire (7.5 cm) as an auxiliary electrode, and LIG-nPt as the working electrode. Raw CV data were exported from MultiTrace software (PalmSens) as csv files for further analysis. Details are available in our published protocol^47^.

### Analysis of voltammograms

**Figure 2:**
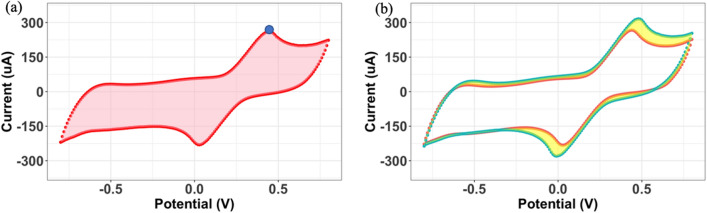
Representative plots of the last sweep from 3 electrodes. Different colors represent the CV curves (final sweep) from different electrodes. (**a**) The polygon (area) between the cathodic and anodic curve (ABC; pink shaded area) and oxidation peak (blue dot). (**b**) Yellow area represents the non-overlapping area between two CV curves (parameter $D$).

LIG electrode quality was defined by the following attributes: (i) batch-to-batch variation of voltammogram shapes, (ii) peak cathodic current, ${I}_{pox}$, and (iii) area between anodic/cathodic curves (ABC, i.e., charge density). Electrodes that demonstrated relatively superior performance in these attributes within a batch were classified as “high-quality” electrodes. The ABC (see representative voltammogram in Fig. 2a**)** was calculated by using the left rectangle method and the peak cathodic current was estimated by grid search. Within this QC process, we assumed that when these two variables (ABC and ${I}_{pox}$) are relatively high, this response signifies improved electroanalytical signal-to-noise ratio, implying reduced random noise influence on the signal.

Figure 2b shows representative voltammograms with different peak currents. The non-overlapping region area between these two CV curves (*D*, see Fig. 2b), was used as the measure of shape similarity. This parameter (*D*) was applied either for comparing multiple repeated CV curves from an individual LIG electrode, or for comparing curves from different LIG electrodes in the same batch. For example, $D({{\varvec{y}}}_{j,k},{{\varvec{y}}}_{{j}{\prime},k})$ represents the non-overlapping region area between CV curves from electrodes *j* and ${j}{\prime}$ in sweep *k*. Here, ${{\varvec{y}}}_{j,k}$ denotes the current (in microamperes, $\mu A$) vector in the CV curve for the $j$ th electrode $k$ th sweep, which is used as the inputs to calculate *D.* Mathematically, *D* is the *symmetric set difference* between the polygons defined by the CV curves from electrodes *j* and ${j}{\prime}$. More details about the calculation of $D$ for two specific CV curves are presented in the Supplementary Materials S1. If the value of *D* is small, one may conclude that these two electrodes will perform similarly for the types of electroanalytical sensing in this study (e.g., development of a capacitive aptasensor).

The exemplified differences in CV curves (polygons) from different electrodes are a common challenge in LIG biosensors development, which demonstrates the need for a QC process to select suitable working electrode replicates. Although not utilized here, techniques such as fast scan CV^48^ can significantly increase the throughput. Analysis of voltammograms from CV can be simple (as is the case here), but the data also contain a wealth of additional material related to process kinetics and reaction rate(s) for further analysis.

### Precheck for each individual electrode

The first step in the data analysis workflow was to determine whether each individual LIG working electrode met the established qualitative criteria for electrochemical behavior, which included generating a characteristic voltammogram for a reversible redox couple during a CV scan with well-defined oxidation and reduction peaks^49,50^. Given that carbon electrodes are known to require multiple voltametric sweeps for surface conditioning^46,51^, a preliminary analysis was conducted to determine the number of repeated sweeps required for convergence. In the electrode assembly used for this study, at least seven voltametric sweeps were required for conditioning a new LIG working electrode. Based on this, we assumed that the observed CV curves from last three sweeps reflected the real values, albeit with inherent random error (with mean zero). If the random error among replicates in the last three sweeps was relatively large, the observed CV curves may be far away from the real value. Therefore, using of data from the last sweep to characterize the electrode behavior would result in increased variance. The first two tasks of the QC process are to: (1) check whether the CV data have successfully converged to a stable result by conducting the convergence diagnostics, and (2) measure the relative error among replicates in the last three sweeps by conducting a replicate error check.

#### Convergence diagnostics

The shape of CV curves between sweeps *k* and $k+1$ were compared to check the stability of outputs from $j$th electrode^52^, which was accomplished by calculating the ratio of $D({{\varvec{y}}}_{j,k},{{\varvec{y}}}_{j,k+1})$ to $AB{C}_{j,k}$ (area between the cathodic and anodic CV curve for electrode *j* sweep *k*):

$${r}_{j,k}=\frac{D({{\varvec{y}}}_{j,k},{{\varvec{y}}}_{j,k+1}) }{AB{C}_{j,k}},$$

where $k=\mathrm{1,2},\cdots K-1$, $K$ is total the number of sweeps. Heuristically, if the ratio ${r}_{j,k}$, which measures the relative change between sweep $k$ and $k+1$, converges to less than or equal to 5%, one may empirically conclude that outputs from the electrodes are stable, and the electrode itself is qualified for manufacturing biosensor.

#### Replicate error check

CV curves from the last three sweeps were compared with the average value for the last three sweeps to measure the replicate error after the conditioning procedure for each electrode presented earlier. Our objective was to determine if relying solely on the curve from final sweep would provide an accurate representation of an electrode performance. To measure the replicate error, for electrode $j$, we started the analysis by computing the average of CV curve ($\overline{{{\varvec{y}} }_{j}}$, the current vector in microampere) derived from the final three sweeps, expressed as

$$\overline{{{\varvec{y}} }_{j}}=\frac{{{\varvec{y}}}_{j,K-2}+{{\varvec{y}}}_{j,K-1}+{{\varvec{y}}}_{j,K}}{3}.$$

Subsequently, we calculated the area of the non-overlapping region, $D({{\varvec{y}}}_{j, k},\overline{{{\varvec{y}} }_{j}})$, between each of the last three sweeps and the average curve for $k=K-2,K-1,K$. We also computed the area between the average curve (ABC) for $\overline{{{\varvec{y}} }_{j}}$, denoted as $AB{C}_{j,\overline{{{\varvec{y}} }_{j}}}$. Finally, we employed the relative error ratio, $D({{\varvec{y}}}_{j, k},\overline{{{\varvec{y}} }_{j}})/AB{C}_{j,\overline{{{\varvec{y}} }_{j}}}$, as a metric. This ratio quantifies the replicate error, which we then compared against a predetermined threshold (5%) to determine the adequacy of the replicate error check.

If the electrode failed the convergence diagnostics (i.e., if ${r}_{j,k}>0.05$), we treated the sample as unqualified electrode and discard it. Additionally, if the relative error ratio was larger than the threshold, we used the average of the last three sweeps to estimate the real value of electrode output and used it as an input to run the subsequent analysis. Otherwise, the CV curves from last sweep were used as inputs in the following analysis.

### Analysis of electrode batches

#### Clustering analysis

Clustering LIG electrodes into several groups based on pairwise similarity via hierarchical clustering analysis is critical for identifying electrodes with similar characteristics. This technique standardizes the quality of biosensors manufactured and minimizes variability in diagnostic testing. In addition, clustering facilitates the detection of outliers within a batch of electrodes, enhancing the overall reliability of the manufacturing process. The inputs of the hierarchical clustering were CV curves from the last sweep for each electrode, assuming low replicate error. Otherwise, the average of CV from last three sweeps were used. The *D* parameter was used as the distance metric in hierarchical clustering to evaluate the pairwise similarity between CV curves. Here, the value of non-overlapping region area $D$ was not normalized by the ABC because the pairwise similarity was compared within the electrodes rather than with a fixed threshold.

To interpret the hierarchical clustering results, we separated the electrodes using a dendrogram approach (hclust function in R). The observations which were far away from the other samples in the hierarchical result were viewed as outliers. Consequently, within each cluster, the electrodes displayed comparable CV shapes, suggesting that the device-to-device variation within the same cluster was relatively smaller than global variation. Subsequently, we conducted a ranking analysis to identify the electrodes most suitable for biosensor manufacturing. These electrodes were expected to exhibit stronger signal power. To do this, we ranked the clusters based on the analysis of peak cathodic current and ABC. Clusters with relatively large peak cathodic current and high ABC were selected. The ranking analysis results were visualized by a heatmap generated with the R program.

#### Selection of replicate LIG working electrodes

Sub-clusters of LIG working electrodes with similar voltammogram shapes, and larger ABC and peak oxidative current were identified using the dendrogram/heatmap visualization. Sub-batches were considered valid if they contained at least three electrodes. During initial development of the QC process (more than 200 individual electrodes tested in batches of 36), a threshold was established for peak oxidation current (200 µA). Electrodes displaying a peak current lower than this threshold were not selected as replicate LIG sub-batches in the development of biosensor for validation of the QC process.

### Validation of QC process for sensing applications

For validation studies, LIG electrodes were fabricated as previously described. Replicates were selected with QC process (treatment group) and without QC process (control group) to fabricate a LIG-based aptasensor. Baseline measurements were used to compare treatment and control groups in our validation studies.

LIG-based aptasensors were fabricated by drop-casting an aptamer suspension onto LIG working electrode surface via streptavidin–biotin coupling method for biofunctionalization step, as described by Moreira et al., 2023. Briefly, LIG-chip electrodes were polarized at + 1.0 V for 600 s, followed by drop-cast of a streptavidin his-tagged suspension (1 mg/ml). The streptavidin-coated LIG-chip electrodes were subsequently drop-cast with an aptamer suspension (1 mg/ml), incubated for 10 min at room temperature, and rinsed with a buffer solution. Following this, the performance of the fabricated aptasensors was characterized using electrochemical impedance spectroscopy (EIS) in an isotonic buffer solution (212.8 mmol/L of NaCl + 64.4 mmol/L of NaHCO_3_) supplemented with 10% (v/v) pooled saliva, pH 8, at 22 °C and 1 atm. The experimental setup for the aptasensors baseline characterization is described in Supplementary Materials S4 and our published protocol^53^.

## Results

In this section, we provide an application of the QC process to the analysis of three unique datasets from LIG electrodes manufactured and tested by biosensor laboratories located at Clemson University (Clemson, SC). In the Supplementary Materials, we discuss the analysis results of our statistical workflow on three additional datasets, including the electrodes manufactured at the Iowa State University (Ames, IA).

The first dataset, used to develop the statistical workflow, consisted of 360 CV curves (each curve contains 320 points) collected from a batch of 36 unique LIG electrodes (ten sweeps for each electrode). These electrodes were all manufactured on the same day by a single operator. Then, to validate the versatility and effectiveness of our QC process, we applied the workflow to two datasets, each consisted of 36 electrodes prepared as a single batch by an individual but on different days. As a proof of concept, the selected electrodes from these two datasets were further used to inform the manufacturing of LIG-based aptasensors. The aptasensor performance was compared with a control group which did not use the QC process (statistical workflow).

The fourth and fifth datasets (see Supplementary Materials S2) consisted of CV curves with the same CV testing setup but were prepared by different operators in the same lab. The sixth dataset (see Supplementary Materials S3) consists of CV curves collected with a different laser system, and a different CV experimental setup (i.e., the data structure differs from the other datasets) at a different laboratory located at Iowa State University (Ames, IA). The purpose of analyzing these three datasets was to evaluate the workflow performance under different scenarios.

### Analysis of individual LIG electrodes

**Figure 3:**
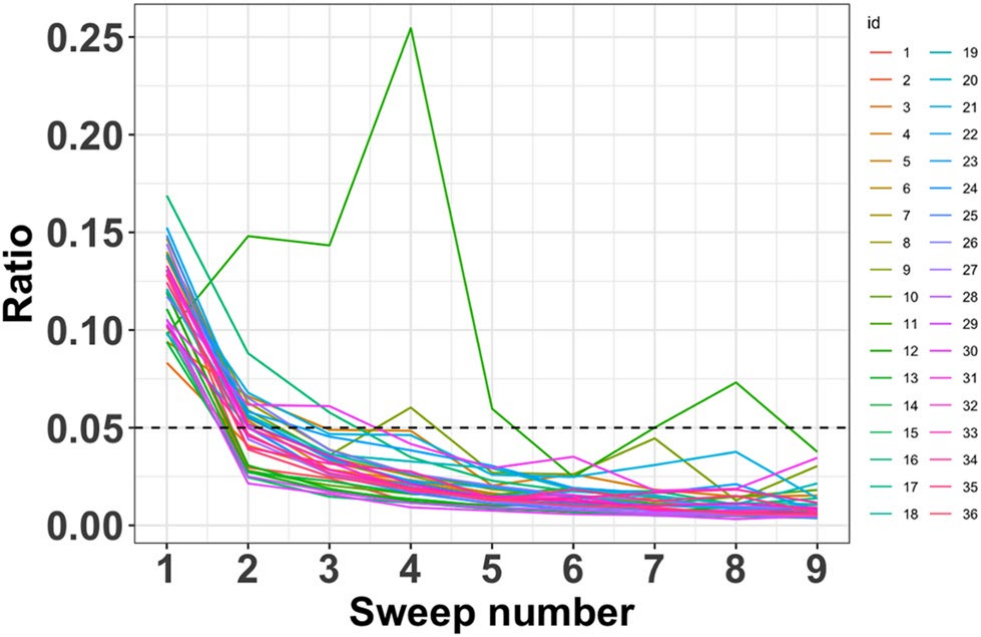
Ratio $({r}_{j,k})$ from convergence diagnostics for a batch of 36 LIG working electrodes (dataset 1). The x-axis is the number of CV sweeps; each line represents the calculated value of the ratio. Independent line plots are shown in the Supplementary Fig. S2.

A representative CV curve of the final sweep taken from dataset 1 is shown in Supplementary Fig. S1. All CV curves display a characteristic reversible redox shape indicating pseudo-capacitive behavior, which is expected in the ferrocyanide/ferricyanide solution. The ratio (${r}_{j,k}$) for convergence diagnostics was calculated for each of the 36 electrodes in dataset 1. Most of the electrodes exhibited ${r}_{j,k}$ values less than 2% (Fig. 3), indicating that the tested electrodes provided stable outputs after ten successive CV sweeps. However, one of the 36 electrodes (number 11) did not converge, displaying a higher ratio compared to the other electrodes (Fig. 3) This suggested that electrode 11 may be a potential outlier, warranting further clustering analysis for confirmation, see subsection analysis of LIG electrodes batches for more details.

Next, a replicate error check was conducted (see Supplementary Table S1 online for details). The relative error ratios ($D({{\varvec{y}}}_{j, k},\overline{{{\varvec{y}} }_{j}})/AB{C}_{j,\overline{{{\varvec{y}} }_{j}}}$) show that the last three CV sweeps had values of less than the 2% threshold for 97% of the electrodes. The exception was electrode number 11, which was consistent with the findings of the convergence diagnostics. This combination of convergence and replicate error checking demonstrates the stepwise process for identifying potential outliers. Additionally, the results confirm that using the last CV sweep to estimate the behavior did not induce bias (indicating behavior was stable after ten successive sweeps).

### Analysis of LIG electrode batches

**Figure 4:**
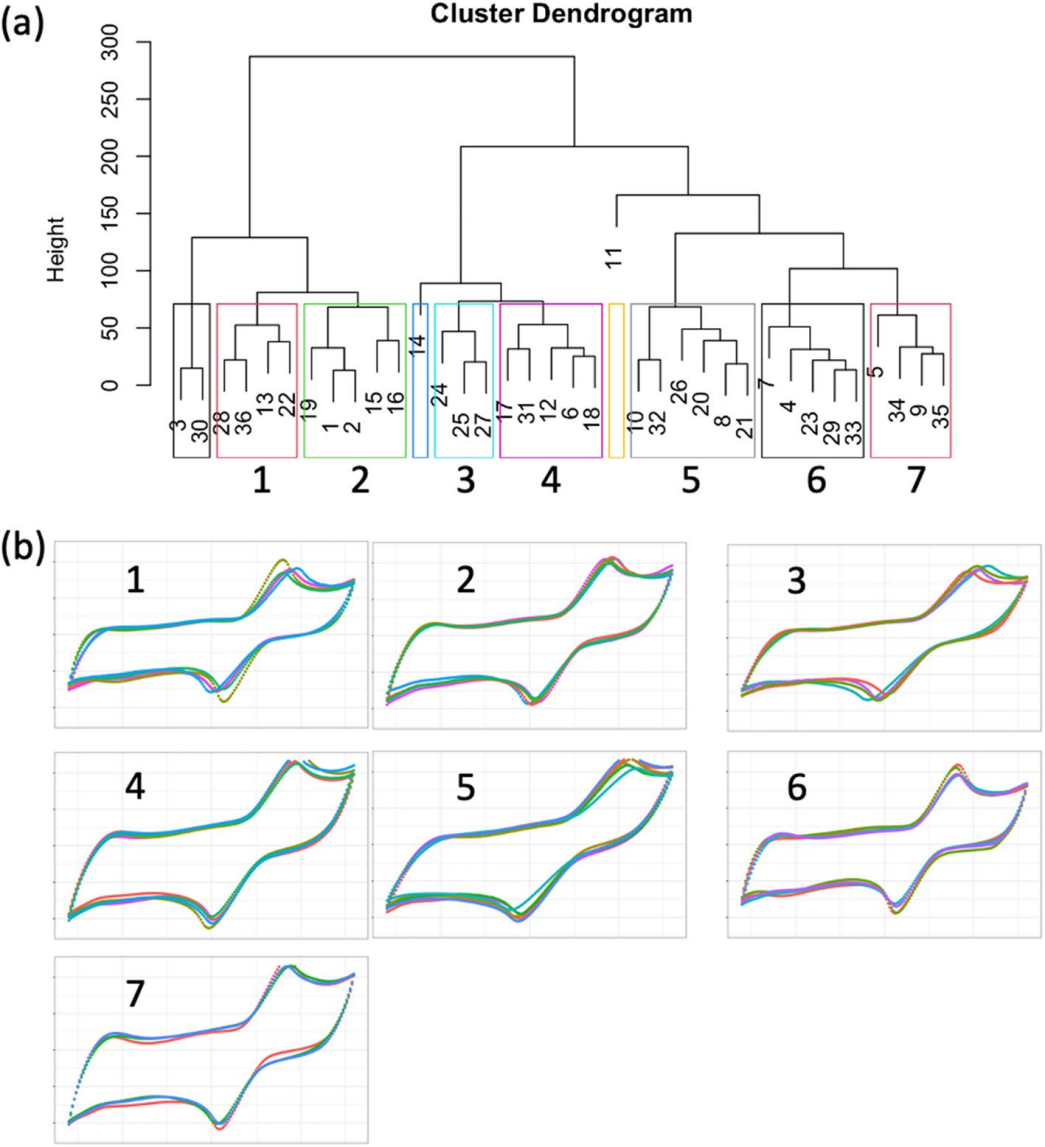
Clustering analysis output for LIG electrodes. (**a**) Representative dendrogram of hierarchical clustering output. Seven clusters that had at least 3 electrodes were highlighted. The sample ID for each LIG electrode is shown at the bottom of the dendrogram. Cluster number is noted below each colored rectangle. (**b**) CV curves from selected electrodes grouped in the clusters analysis showed in panel as. The selected clusters contained at least 3 electrodes. Cluster numbers correspond with the labeling of colored rectangles in panel a.

Clustering analysis was conducted to split the electrodes to several groups based on their pairwise similarity. The clustering result is visualized in Fig. 4 using a dendrogram, where each cluster is indicated with color rectangles and numbered. The dendrogram clearly showed that electrode 11 was separated from the other electrodes, visualizing the conclusions in section "Analysis of individual LIG electrodes" regarding outlier screening. Applying the workflow for selection of replicate electrodes, ten clusters were identified by the clustering algorithm, but three of them did not contain a valid triplicate and were therefore discarded. The seven valid clusters are labeled in Fig. 4a, and the corresponding CV curves for the groups with at least three similar electrodes are visualized in Fig. 4b. Notably, the CV curves within each cluster group shared a similar voltammogram shape, indicating that the electrodes in each cluster had similar behavior. For a more comprehensive view of the CV curves in each cluster, refer to Supplementary Fig. S3.

**Figure 5:**
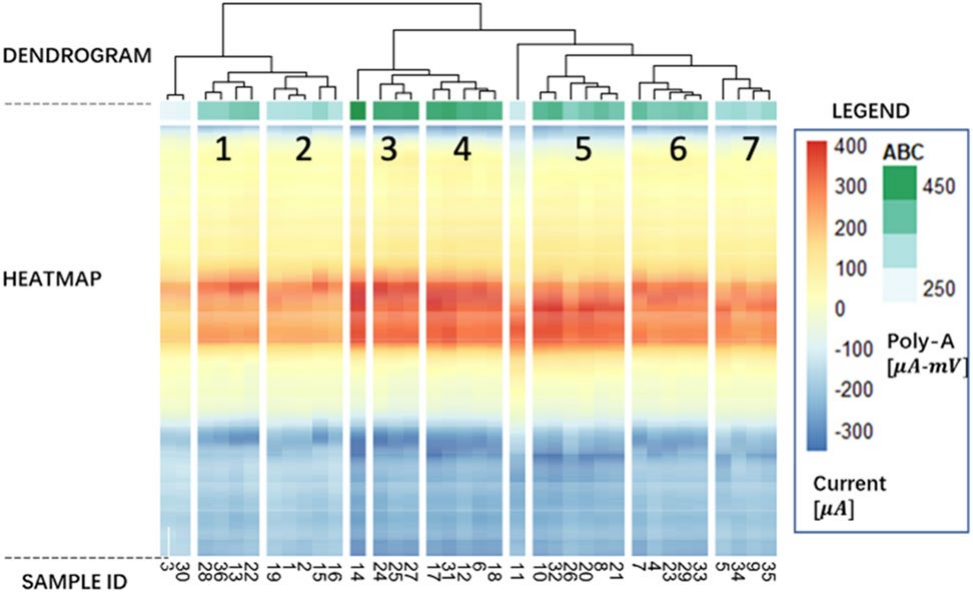
Representative heatmap based on key electrodes features (ABC and peak cathodic current).

Supplementary Table S2 shows the average ABC and average peak cathodic current for each cluster shown in Fig. 4. Cluster 3 (electrodes 24, 25 and 27) had the highest ABC and peak current, while cluster 7 (electrodes 5, 34, 9 and 35) had the lowest values for both variables. A heatmap was overlaid on the dendrogram to visualize results from clustering and ranking analysis (Fig. 5). The heatmap consists of three components: (i) the dendrogram described in Fig. 4a for visualizing clustering analysis results, (ii) an independent heatmap for visualizing ABC of each cluster (green colorscale), and (iii) an independent heatmap for visualizing average peak cathodic current (red colorscale). In this analysis (dataset 1), electrodes in the 3rd and 4th clusters exhibited both large ABC area and peak cathodic current, indicated by the dark green and red relative to other clusters.

### Validating the QC process

**Figure 6:**
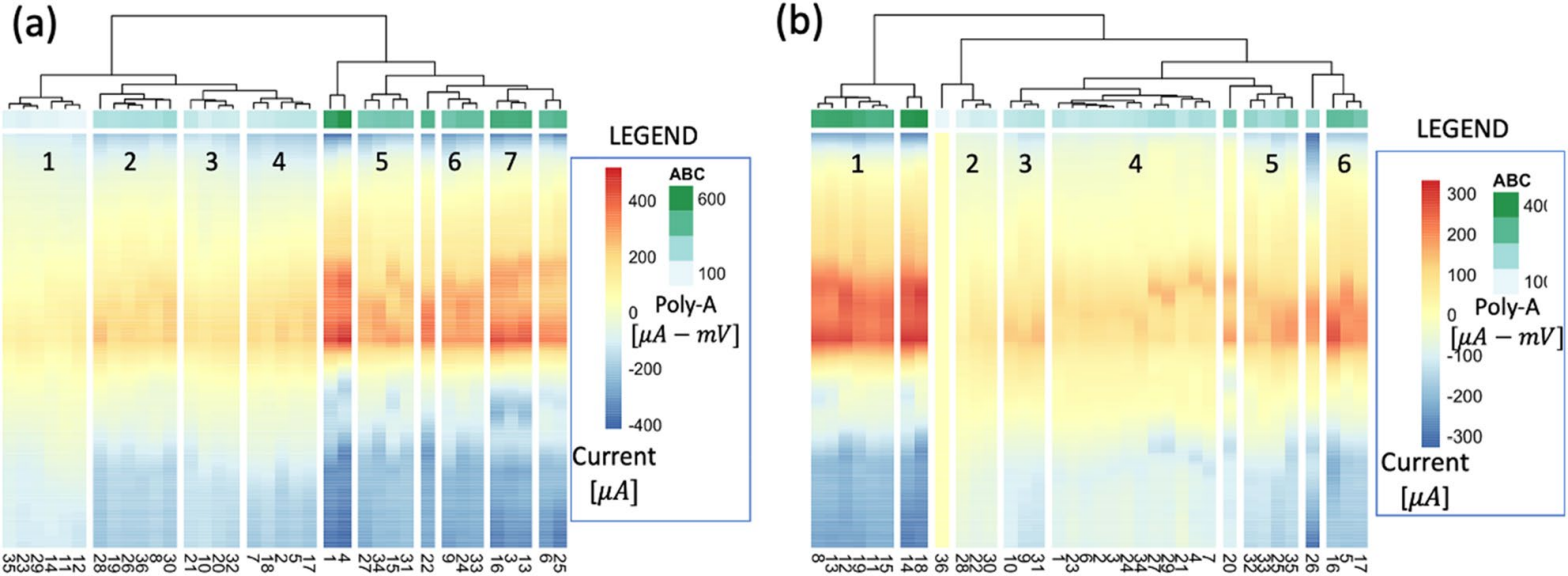
Heatmaps from LIG electrodes in dataset 2 and 3. (**a**) The heatmap of key features of electrodes from dataset 2. There are seven clusters which contain at least three electrodes (labeled by number). By comparing the ABC (green bar) and peak cathodic current (red area), the selected three triplicate electrodes are electrodes in 5th, 6th, and 7th clusters, respectively. (**b**) The heatmap of key features of electrodes from dataset 3. There are six clusters which contain at least three electrodes (labeled with the corresponding integers). By comparing the ABC (green bar) and peak cathodic current (red area), we selected two triplicate electrodes in cluster 1 and one triplicate electrode in cluster 6.

The effectiveness of this developed process was validated by comparing the performance of manufactured biosensors with (treatment group) and without (control group) the use of the QC process depicted herein. Four triplicates (n = 12) were chosen by a highly skilled-lab researcher via naked eye inspection of CV curves in the control group. In the treatment group, another two independent batches of electrodes were prepared using the same experimental protocol. Notably, in both datasets for this validation study, all electrodes passed the pretest, hence all were considered for the selection stage. The outcomes of the selection process for both datasets are depicted in Fig. 6. Based on the selection statistical workflow findings (illustrated by the dendrogram and heatmap), three triplicates were chosen from each dataset (n = 18).

The chosen electrodes, irrespective of whether they were chosen using naked eye inspection or the selection program, were utilized for development of an LIG-based aptasensor as previously described in our previous work^46^. The primary output from the aptasensors, the Bode impedance curve, served as the benchmark for baseline evaluation. Imaginary impedance (Z'') at a cutoff frequency of 0.1 Hz to 0.01 Hz was used to analyze biosensors from both treatment and control groups (see Supplementary Fig. S4 and Supplementary Table S3). In this study, we only compared the outputs for the aptasensor baseline characterization in a buffer solution to demonstrate use of the QC process in screening LIG electrode batches. Further studies are required to study the electrode behavior in virus detection.

Principal component analysis (PCA) was applied for analyzing Z’’ at a cutoff frequency of 0.1 Hz to 0.01 Hz (both treatment and control groups), we adopted a data analysis method from our prior study^46^. Scatter plots of the first two principal components (PCs) derived from the PCA are shown in Supplementary Fig. S5. The first PC was chosen to represent the raw data (i.e., the imaginary impedance) as it accounted for 99% of the sample variance. The standard deviation (SD) of the first PC served as a metric to assess the measurement variation of the aptasensors, reflecting the reproducibility of repeated apatsensors for diagnostic testing. Aptasensors within the treatment group demonstrated 13% reduction in SD relative to the control group (SD = 2.450 treatment, SD = 2.757 control), indicating a smaller measurement variation.

## Discussion

QC is an important step in the development of sensor technology. The quality of LIG electrodes can directly influence the quality of data collected and, thus, the performance of a biosensor-based system. Higher quality and consistency in the selection of electrodes result in lower biosensor measurement variation. In this manuscript, we introduced a method to incorporate statistical QC into the design and production of biosensors. The developed QC process was applied in three independent biosensor studies, and the results show that the proposed procedure allows one to select similar and functional electrodes from a batch of samples. Additionally, using the developed QC process can reduce biosensor variability, providing moderate improvement in terms of the reproducibility over the selection by a highly experienced operator. Other techniques have been used to study carbon electrode quality in the manufacturing process, such as Raman spectroscopy and nanoscale metrology^43,44^, but this manuscript is the first to create a statistical workflow for QC.

The designed QC statistical workflow can effectively split electrodes into distinct clusters based on pairwise similarity within a single dataset, thereby pinpointing the high-quality electrodes for biosensor production. However, combining datasets, especially those with increased variability due to biological differences or distinct operators (elaborated in the Supplementary Materials S2), is not recommended for this workflow. To navigate this challenge, more sophisticated statistical approaches, such as mixed effects models, are essential. These could incorporate operator-specific information as a random effect in the model.

Based on the study of datasets, two types of outliers were found: (1) electrodes that show significantly lower functionality (smaller ABC and peak cathodic current) than the others (e.g., the 11th electrode in the dataset 1); (2) electrodes that show significantly higher functionality (higher ABC and peak cathodic current) than others. Addressing the first category is straightforward; such underperforming outliers can be readily discarded. The challenge arises with the second category. While these superior-performing electrodes deviate from the majority, they possess high-quality results in biosensor baseline performance. Our suggestion is to retain these electrodes for future applications, aiming to pair them with upcoming electrodes that align with their performance profile.

The code we developed for our analysis can be accessed on our GitHub repository. It is readily applicable for LIG electrodes with an identical CV testing setup and can be easily adapted for data from varying CV configurations. A single test round suffices to pinpoint the optimal electrodes, rapidly offering crucial information for electrode selection support. Therefore, the developed QC process in this study enhances the automation LIG electrode fabrication workflow. We recommend using electrodes within the clusters with high average ABC and peak cathodic current to fabricate LIG-based biosensors. Biosensors constructed using these select electrodes will likely deliver signals with minimal variation. Nonetheless, we acknowledge that electrodes not chosen for these premium clusters still have value. Assuming these electrodes have surpassed preliminary testing and demonstrated comparable performance, they could be reserved for less critical applications, such as student training exercises. Implementing such a strategy ensures efficient resource allocation while maximizing experimental outcomes.

Using the developed QC process introduced in this manuscript enables the selection of similar and functional electrode from a batch, optimizing the manufacture of biosensors and minimizing measurement variation. However, the real-world applications revealed instances where the finalized biosensors still exhibited large data variations despite the support from QC process. We hypothesize that this variation arises from subsequent stages like biofunctionalization and sample detection.

To mitigate this variability, there are two viable strategies: increasing the number of biosensor replications or integrating more advanced statistical calibration during data analysis. While augmenting replication improves the detection results quality, it may also increase operational costs. Techniques from statistical and computational disciplines are often used to assess data uncertainty. A direct method involves considering the biosensor ID as a random effect in models, accounting for variations across different batches.

Although not studied in detail here, this technique is also useful for future studies focused on electrode reusability. In some applications (e.g., pathogen detection as shown here), diagnostic tests are not reusable due to the risk of contamination. In this instance, the QC process shown here may be limited to large batch fabrication efforts. However, in other applications (e.g., monitoring nutrients in water or non-hazardous chemicals), the technique could be used to study material regeneration, signal hysteresis and/or device reuse. Regardless, high quality LIG electrodes are urgently needed to ensure accuracy of diagnostic testing.

## Conclusions

The use of data science methods is anticipated to become increasingly prevalent in the field of sensor research and development. In this manuscript, we develop for the first time a rapid and user-friendly QC process designed to identify similar and functional electrodes from a large fabrication batch. This, in turn, facilitates the production of biosensors expected to exhibit characteristics such as low measurement variation and substantial signal power.

### Supplementary Information

Supplementary Information.

## Data Availability

The data that support the findings of this are openly available in Zenodo communities and Github at: https://zenodo.org/communities/qclig; https://github.com/qqqqqhanyu/Example-code-and-R-program-for-electrode-QC-process.
