# Supplementary material for: Improving high throughput manufacture of laser-inscribed graphene electrodes via hierarchical clustering
Source: Sci Rep. 2024 Apr 5;14:7980. doi: 10.1038/s41598-024-57932-z (PMC10995179; doi:10.1038/s41598-024-57932-z)
Supplement: Supplementary file 1 — Supplementary Information. [file 41598_2024_57932_MOESM1_ESM.docx]

Improving high throughput manufacture of laser-inscribed graphene electrodes via hierarchical clustering

Hanyu Qian^a^, Geisianny Moreira^b^, Diana Vanegas^c^, Yifan Tang^d^, Cicero Pola^e^, Carmen Gomes^e^, Eric McLamore^b,c*^, Nikolay Bliznyuk^a,f*^

^a^ Department of Agricultural and Biological Engineering, University of Florida, Gainesville, FL 32611, USA

^b^ Department of Agricultural Sciences, Clemson University, Clemson, SC 29634, USA

^c^ Environmental Engineering and Earth Sciences Department of Engineering, Clemson University, Clemson, SC 29634, USA

^d^ Department of Plant and Environmental Science. Clemson University, Clemson, SC 29634, USA

^e^ Department of Mechanical Engineering, Iowa State University, Ames, IA 50011, USA

^f^ Departments of Statistics, Biostatistics and Electrical & Computer Engineering, University of Florida, Gainesville, FL 32611, USA

^*^ Corresponding authors: [emclamo@clemson.edu](mailto:emclamo@clemson.edu), [nbliznyuk@ufl.edu](mailto:nbliznyuk@ufl.edu)

**Supplementary Tables**

Supplementary Table S1. The replication variance of each biosensor

| sample id | rep 8 | rep 9 | rep 10 | Average |
| --- | --- | --- | --- | --- |
| 1 | 0.0062 | 0.0023 | 0.0062 | 0.0049 |
| 2 | 0.0033 | 0.0024 | 0.0040 | 0.0033 |
| 3 | 0.0085 | 0.0041 | 0.0070 | 0.0065 |
| 4 | 0.0083 | 0.0029 | 0.0091 | 0.0068 |
| 5 | 0.0119 | 0.0057 | 0.0121 | 0.0099 |
| 6 | 0.0049 | 0.0028 | 0.0053 | 0.0043 |
| 7 | 0.0064 | 0.0019 | 0.0071 | 0.0051 |
| 8 | 0.0083 | 0.0005 | 0.0080 | 0.0056 |
| 9 | 0.0110 | 0.0123 | 0.0196 | 0.0143 |
| 10 | 0.0145 | 0.0035 | 0.0159 | 0.0113 |
| 11 | 0.0465 | 0.0318 | 0.0213 | 0.0332 |
| 12 | 0.0044 | 0.0004 | 0.0041 | 0.0030 |
| 13 | 0.0078 | 0.0020 | 0.0062 | 0.0053 |
| 14 | 0.0053 | 0.0049 | 0.0042 | 0.0048 |
| 15 | 0.0048 | 0.0006 | 0.0048 | 0.0034 |
| 16 | 0.0102 | 0.0036 | 0.0117 | 0.0085 |
| 17 | 0.0055 | 0.0008 | 0.0052 | 0.0038 |
| 18 | 0.0076 | 0.0052 | 0.0080 | 0.0069 |
| 19 | 0.0081 | 0.0085 | 0.0143 | 0.0103 |
| 20 | 0.0061 | 0.0044 | 0.0079 | 0.0061 |
| 21 | 0.0256 | 0.0130 | 0.0156 | 0.0181 |
| 22 | 0.0083 | 0.0014 | 0.0081 | 0.0059 |
| 23 | 0.0148 | 0.0071 | 0.0091 | 0.0103 |
| 24 | 0.0052 | 0.0013 | 0.0052 | 0.0039 |
| 25 | 0.0047 | 0.0007 | 0.0042 | 0.0032 |
| 26 | 0.0093 | 0.0007 | 0.0091 | 0.0064 |
| 27 | 0.0051 | 0.0007 | 0.0048 | 0.0035 |
| 28 | 0.0046 | 0.0016 | 0.0052 | 0.0038 |
| 29 | 0.0034 | 0.0014 | 0.0040 | 0.0029 |
| 30 | 0.0069 | 0.0170 | 0.0183 | 0.0141 |
| 31 | 0.0053 | 0.0037 | 0.0059 | 0.0050 |
| 32 | 0.0081 | 0.0038 | 0.0067 | 0.0062 |
| 33 | 0.0059 | 0.0017 | 0.0060 | 0.0045 |
| 34 | 0.0120 | 0.0078 | 0.0085 | 0.0095 |
| 35 | 0.0116 | 0.0038 | 0.0083 | 0.0079 |
| 36 | 0.0057 | 0.0009 | 0.0056 | 0.0040 |

The values in Supplement Table S1 column 2 to 4 were the relative error ratios that measure the error between last three sweeps with the average. More details about how to calculate the ratio were introduced in section 2.6.2. The values in last column were the average of column 2 to 4, which measures the replicate error of last three sweeps. Majority of the average ratios were less than 2%, meaning that the replicate variance of each electrode was small.

**Supplementary Table. S2**. The average ABC and peak cathodic current of each cluster

| Cluster | ABC ($\mu A-mV$) | Peak current ($\mu A$) |
| --- | --- | --- |
| 1 | 351 | 304 |
| 2 | 310 | 262 |
| 3 | 426 | 353 |
| 4 | 416 | 354 |
| 5 | 374 | 337 |
| 6 | 365 | 307 |
| 7 | 321 | 276 |

**Supplementary Table S3.** The standard deviation (SD) of signals by frequency

| Freq (Hz) | Control group  SD (n = 12) | Treatment group  SD (n = 18) |
| --- | --- | --- |
| 0.01 | 9326 | 7173 |
| 0.0126 | 7556 | 5902 |
| 0.0158 | 6085 | 4928 |
| 0.02 | 4900 | 4066 |
| 0.0251 | 3947 | 3362 |
| 0.0316 | 3187 | 2833 |
| 0.0398 | 2589 | 2381 |
| 0.0501 | 2118 | 2006 |
| 0.0631 | 1723 | 1671 |
| 0.07943 | 1396 | 1384 |
| 0.1 | 1133 | 1143 |

**Supplementary Figures**


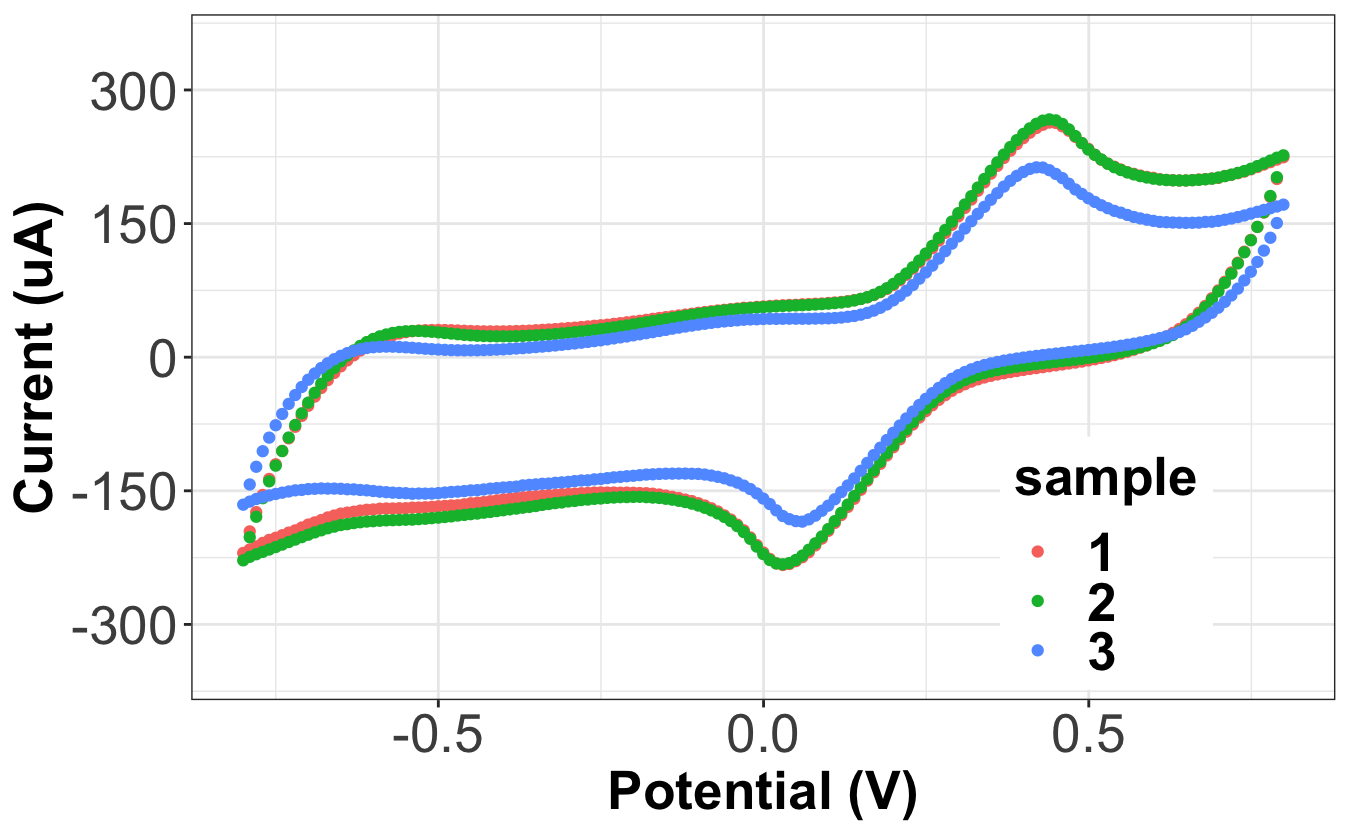


**Figure S1**. Representative CV plots (last CV sweep) for the first 3 electrodes from a batch. Different colors represented the outputs from different electrodes.


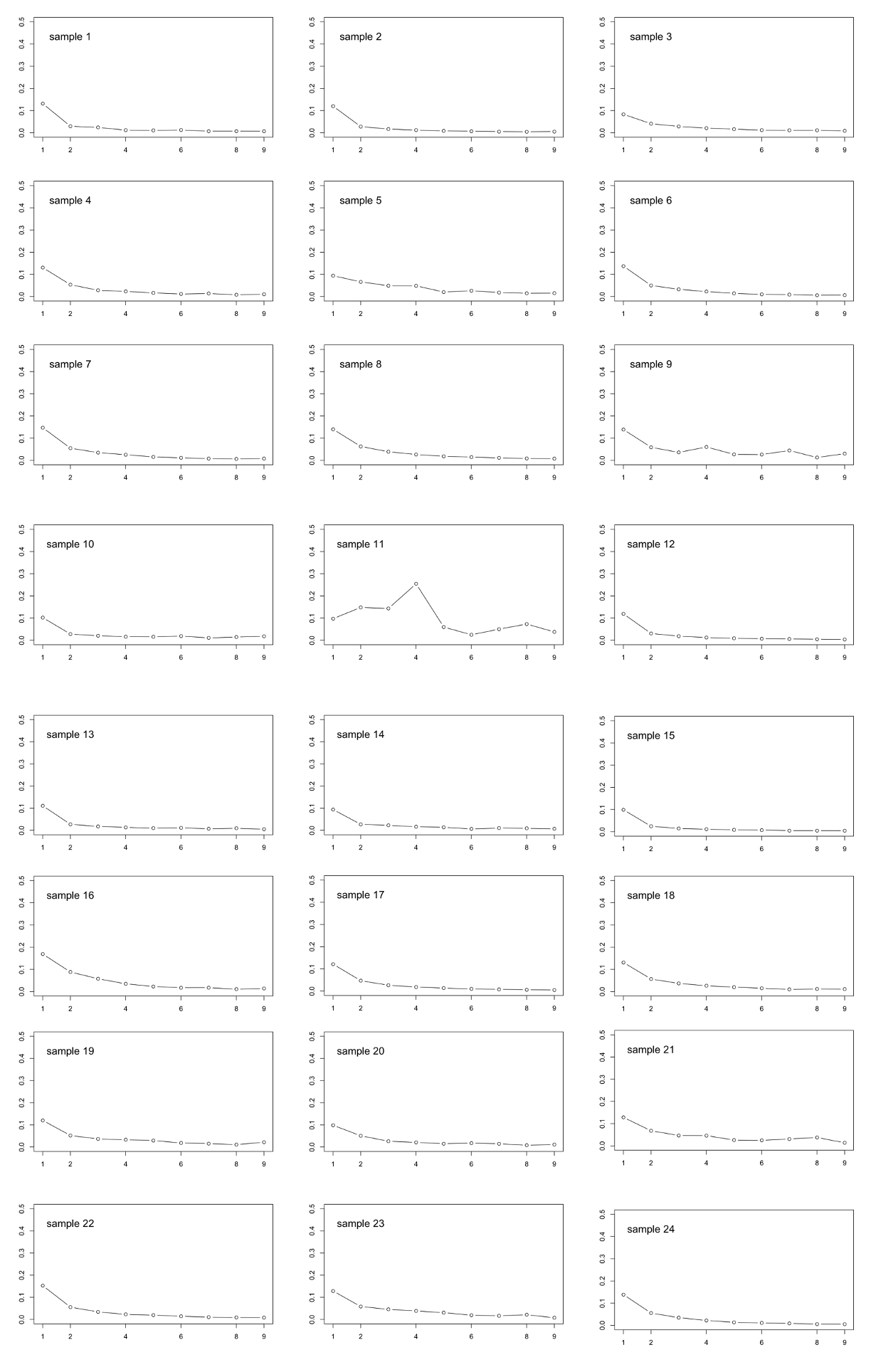


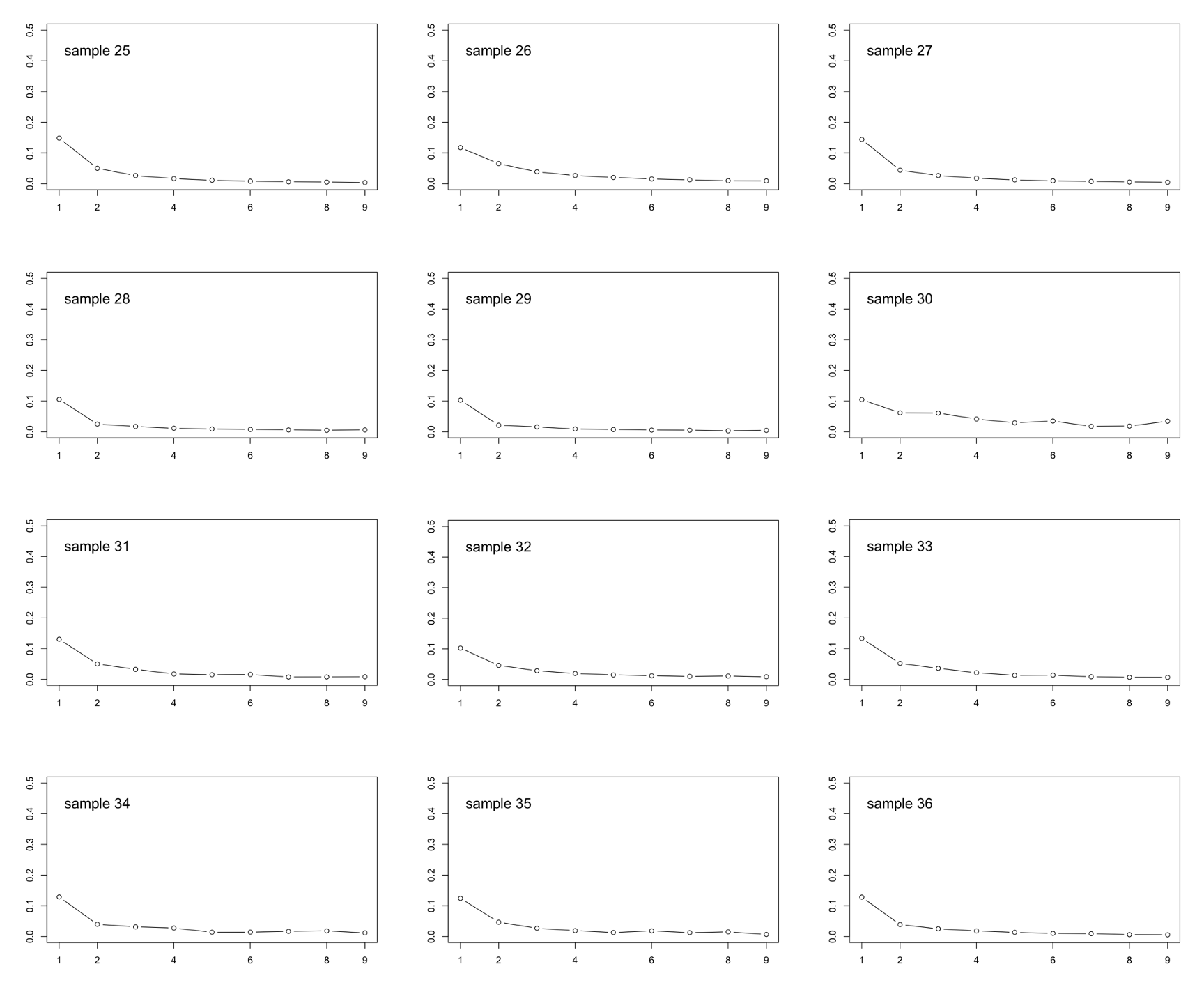


**Figure S2**. Independent line plots of shape change ratio for 36 electrodes in the first dataset. The x-axis is the number of sweeps, and y-axis is the shape change ratio. Apart from the 11st electrode, the tested electrodes converge fast, with shape change ratio less than 5% after six sweeps.


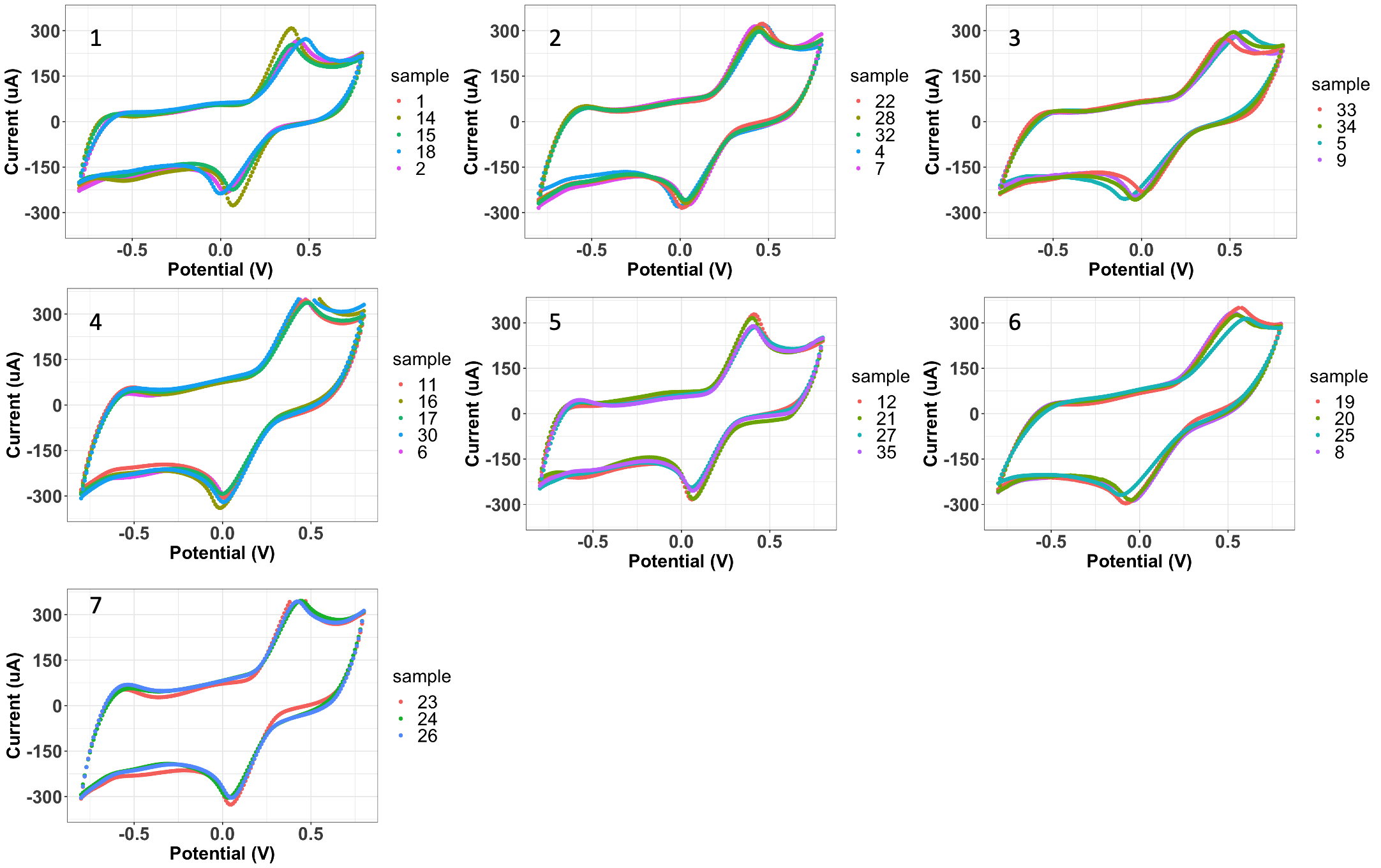


**Figure S3**. Visualizing the electrodes CV curves by clusters which contains at least 3 electrodes


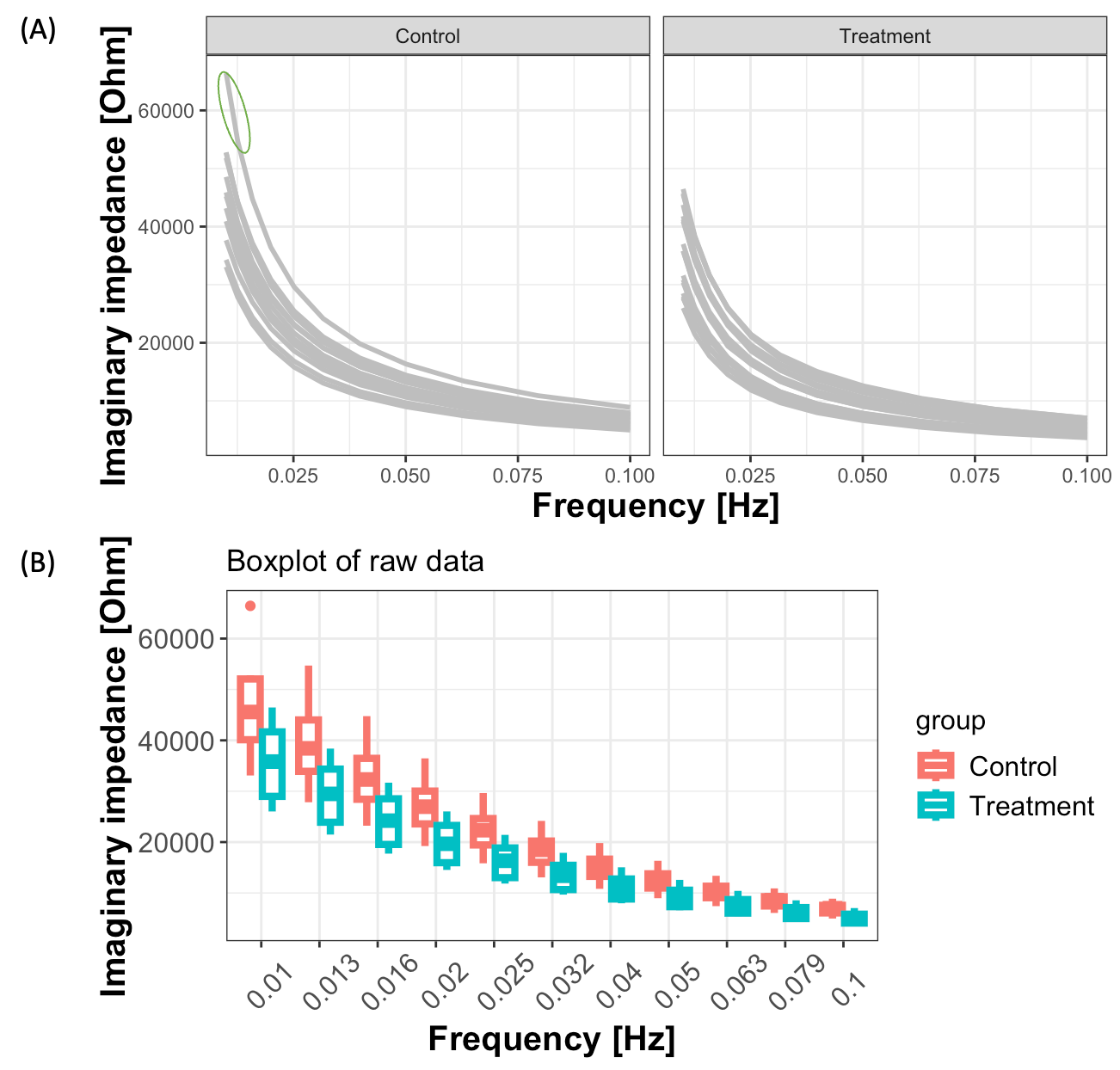


**Figure S4.** The imaginary impedance spectrum of aptasensors for the baseline solution. (A) a plot displaying raw data at the cut-off frequency (i.e., 0.01 Hz to 0.1 Hz) from each group, where each line corresponds to an individual observation (biosensor output curve), and (B) a boxplot of the impedance data. The plots reveal an outlier within the control group. Both panels suggest a potential difference in the mean impedance curves between the treatment and control groups. Using two-way ANOVA with interactions to compare the group means revealed the statistically significant effect of frequency-by-group interaction (F-test p-value less than 0.001). This underscores the importance of “standardization” of sensor outputs in subsequent analyses to ensure exchangeability of the data across multiple operators, manufacturing protocols, sites or operating conditions (referred in current imaging literature as “harmonization” and “registration” and in the microarray literature as “normalization”).


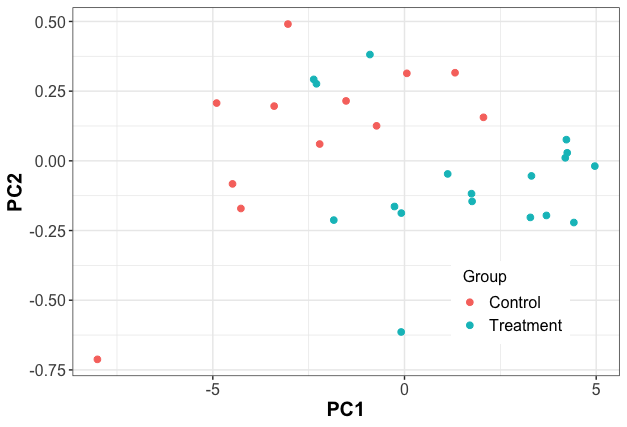


**Figure S5**. PCA graph for distinguish control group (without selection algorithm, n = 12) and treatment group (with selection algorithm, n = 18). There is an outlier in the control group (located at the left bottom), which is not found for treatment group.

**Supplementary S1. Non-overlapping area calculation**

The non-overlapping region area (*D*, see **Fig. 2b**), an extension of absolute difference between two current vectors, was used to measure the similarity between the anodic and cathodic CV curves. The value $I_{j,k,l}$ was defined as the current value from $j$th electrode, $k$th sweep and at point $l$ ($j=1, 2, \cdots J, k=1, 2, \cdots K, l=1, 2,\cdots L$, J is the number of electrodes, $K$ is the number of sweeps, L is the number of points on the CV curve). To estimate the value of $D$, we used the left rectangle method to calculate the area of the non-overlapping region (yellow part in **Fig. 2b);** For example, comparing the CV curves from electrode $j$ and $j'$, sweep *k*):

$D(\boldsymbol{y}_{j,k},\boldsymbol{y}_{j^{'},k})=\sum_{k=1}^{K} {|I}_{j,k,l}-I_{j^{'},k,l}\left| \cdot\Delta\right|U_{l}|$.

Here $\boldsymbol{y}_{j,k}$ is the current vector (in microampere, $\mu A$) for electrode $j$, $k^{th}$ sweep ($\boldsymbol{y}_{j,k}=\{I_{j,k,1},I_{j,k,2}\cdots I_{j,k,L}\}$), ***U*** is the potential vector, $\Delta\left| U_{l} \right|=|U_{l+1}-U_{l}|$, and $U_{L+1}=U_{1}$ (because the CV curve starts and ends at same point). Assuming a consistent CV testing experimental setup within one study, the potential vector ***U*** remains fixed for each CV curve.

**Supplementary S2. Selection results for datasets collected from different operators**

We further explored the performance of our electrodes selection algorithm by testing it with data from multi-operators. The variability of datasets from different operators can be even larger than the variability of dataset from the same operator.

In addition to the three datasets introduced in result section, we included two datasets from different operators. Each of the new datasets included 48 electrodes. The example plots of CV curves from the fourth and fifth dataset are visualized in Supplementary Fig. S6, respectively. We ran our electrodes selection algorithm for these two new datasets separately. In the first step, we conducted the convergence diagnostics and replicate error check introduced in section 2.6. Electrode 31 in the fourth dataset and electrodes 19, 41, 45 in the fifth dataset failed to pass the convergence test. After discarding those unqualified samples, we ran the hierarchical clustering analysis and ranking analysis and heatmaps are visualized in Supplementary Fig. S7.


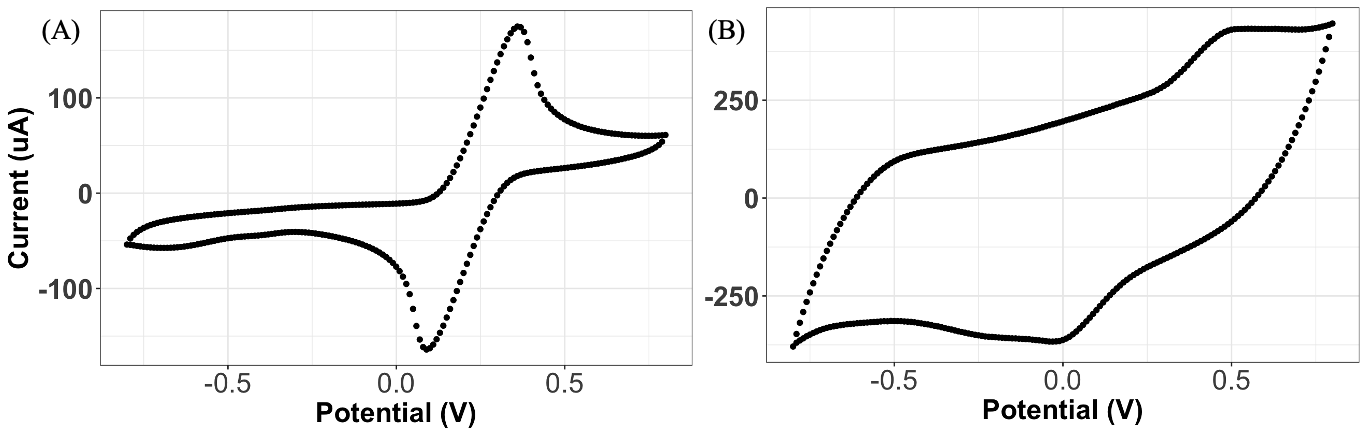


**Figure S6**. The average of electrodes outputs from different operators. The fifth dataset (see **Fig. S6(B)**) shared the same biological features as the dataset in section 2.5. While the fourth one (see **Fig. S6(A)**) had biological difference.


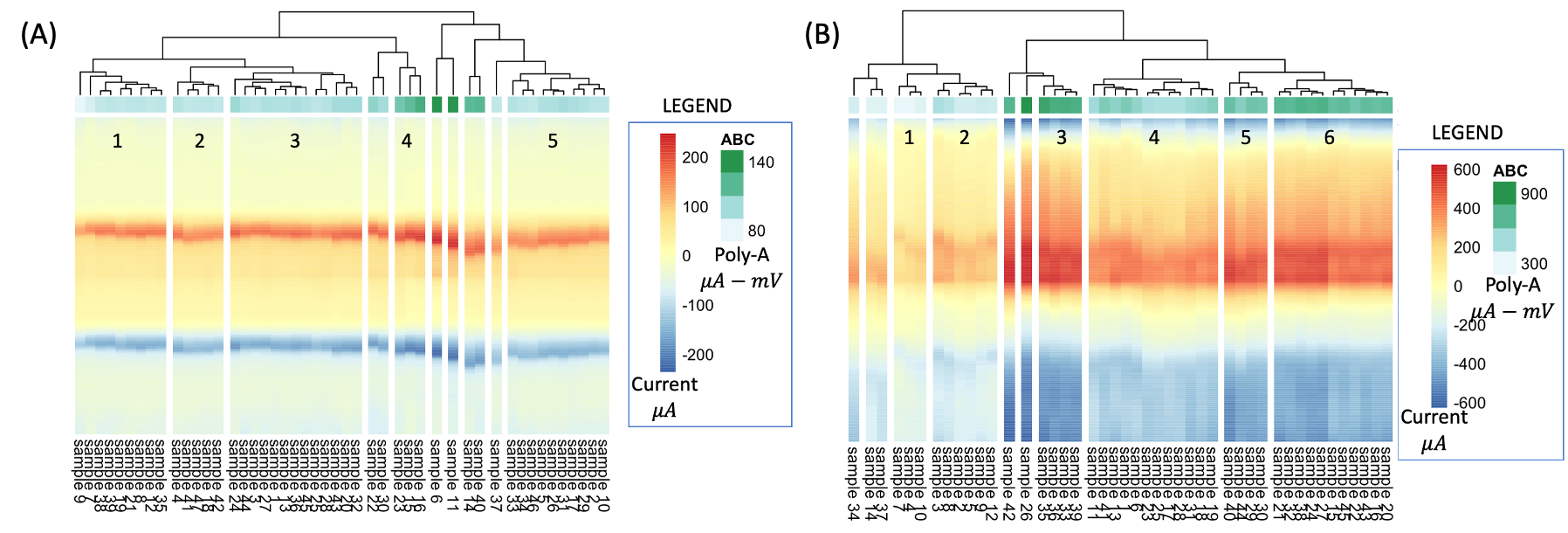


**Figure S7**. The heatmaps of selection results from dataset 4 and 5; (A): The heatmap of key features of electrodes from dataset 5. There are 5 clusters which contain at least three electrodes (labeled by number). By comparing the ABC (green bar) and peak cathodic current (red area), the selected electrodes are electrodes in 4^th^ cluster; (B): The heatmap of key features of electrodes from dataset 5. There are 6 clusters which contain at least three electrodes (labeled by number). By comparing the ABC (green bar) and peak cathodic current (red area), the electrodes in cluster 3, 5, and 6 are better than the rest of electrodes.

**Supplementary S3. Selection results for datasets with different CV testing experimental set up**

In this section, we explore the performance of our selection algorithm by running it with a dataset that has different CV testing setups. The CV testing in this dataset was carried out using a solution of 5 mM Fe(CN)63-/Fe(CN)64- redox probe in 0.1 M KCl, with a potential range from -0.4 to 0.8 V and a sweep rate of 100 mV/s for only one cycle. The new dataset consists of CV curves from 39 Ag/AgCl reference electrodes, and each curve consists of 239 points. Since each electrode was tested only once, we began with the hierarchical clustering analysis. Subsequently, based on the results of the clustering analysis, the ranking analysis was performed, and the results are visualized in Supplementary Fig. S8.


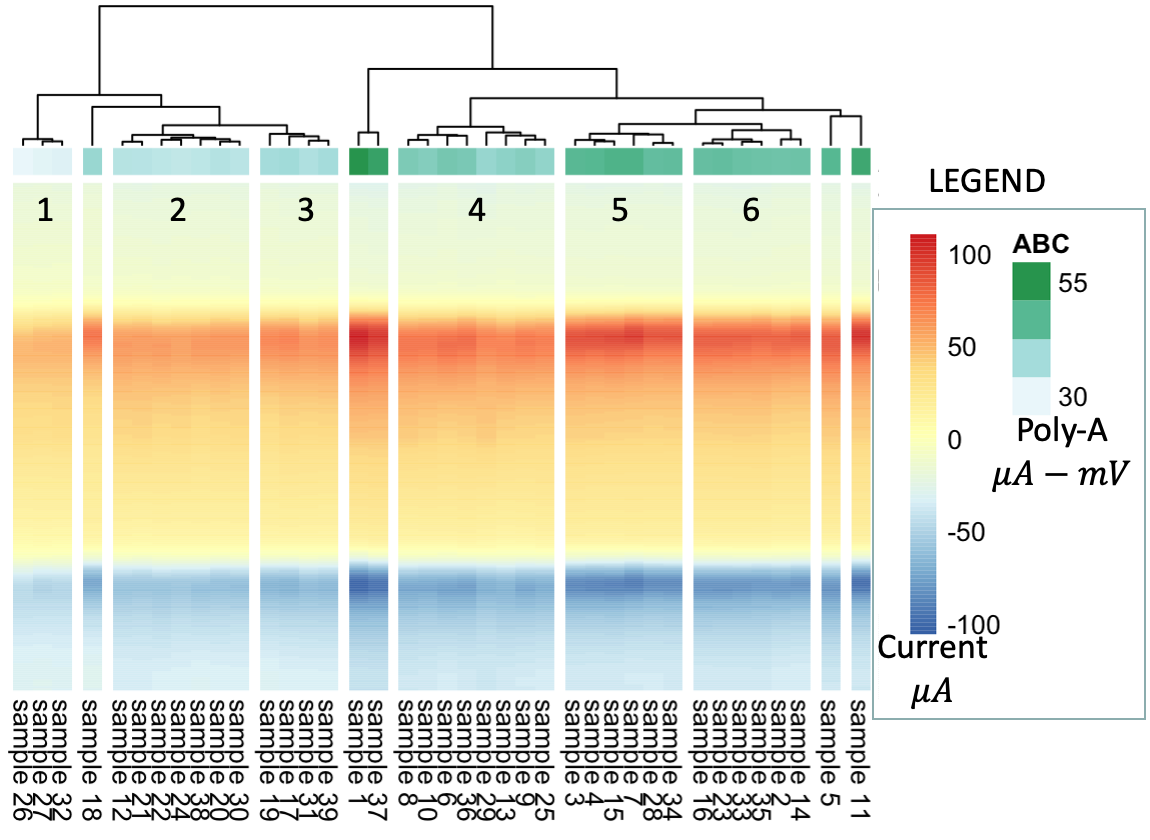


**Figure S8**. The heatmap of key electrodes features. There are 6 clusters which contain at least three electrodes (labeled by number). By comparing the ABC (green bar) and peak cathodic current (red area), the selected electrodes are electrodes in 5^th^ and 6^th^ cluster.

**Supplementary S4. Aptamer-based biosensor baseline characterization for validation study**

Electrochemical Impedance Spectroscopy (EIS) testing was performed for baseline characterization of aptamer-based biosensor. Electrochemical measurements were performed using a benchtop potentiostat (PalmSens®, MultiPalmSens4, Houten, Netherlands). EIS testing was carried out in a non-Faradaic mode by immersing the biosensors in a physiological solution (212.8 mmol/L of NaCl + 64.4 mmol/L of NaHCO_3_) supplemented with 10% (v/v) pooled saliva, pH 8, at 22°C and 1 atm. EIS settings for all testing were: frequency range of 0.01 Hz–10,000 Hz, AC amplitude of 0.08 V, and a DC voltage of 0.36 V. EIS tests yield data on total impedance (Z), real impedance (Z′), imaginary impedance (Z″), series capacitance (Cs), real capacitance (C′), and imaginary capacitance (C″) at 63 cutoff frequencies within the described frequency range.

**Supplementary S5. Software information**

Universal Control Panel (UCP) software was used to fabricate LIG electrodes in the Universal Laser (VLS2.30DT). MultiTrace4 software for MultiPalmSens4 potentiostat (PalmSens, Houten, Netherlands) was used to acquire signals from electrochemical analysis (cyclic voltammetry, chronoamperometry and electrochemical impedance spectroscopy). Data processing and analysis was performed using the program language R version 4.1.1^1^ with the following packages: “ggplot2” ^2^, “pheatmap” ^3^ and “readxl” ^4^. All written code can be found on

1. R Core Team. R: A Language and Environment for Statistical Computing. Preprint at https://www.R-project.org/ (2021).

2. Hadley Wickham. *ggplot2: Elegant Graphics for Data Analysis*. (2016).

3. Kolde, R. pheatmap: Pretty Heatmaps. Preprint at https://CRAN.R-project.org/package=pheatmap (2019).

4. Wickham, H. & Bryan, J. readxl: Read Excel Files. Preprint at https://CRAN.R-project.org/package=readxl (2019).
